# Levels of outpatient prescribing for four major antibiotic classes and rates of septicemia hospitalization in adults in different US states - a statistical analysis

**DOI:** 10.1186/s12889-019-7431-8

**Published:** 2019-08-19

**Authors:** Edward Goldstein, Scott W. Olesen, Zeynal Karaca, Claudia A. Steiner, Cecile Viboud, Marc Lipsitch

**Affiliations:** 1 Center for Communicable Disease Dynamics, Department of Epidemiology, Harvard TH Chan School of Public Health, 677 Huntington Ave, Kresge Room 506, Boston, MA 02115 USA; 2 U.S. Department of Health & Human Services, Agency for HealthCare Research and Quality, Rockville, MD 20850 USA; 3 Institute for Health Research, Kaiser Permanente Colorado, Denver, CO 80231 USA; 4 Division of International Epidemiology and Population Studies, Fogarty International Center, National Institutes of Health, Bethesda, MD 20892 USA; 5 Department of Immunology and Infectious Diseases, Harvard TH Chan School of Public Health, Boston, MA 02115 USA

**Keywords:** Septicemia, Sepsis, Hospitalization rate, Antibiotic prescribing, Penicillins, Fluoroquinolones, Cephalosporins, Macrolides, African Americans, Daily temperature

## Abstract

**Background:**

Rates of sepsis/septicemia hospitalization in the US have risen significantly during recent years. Antibiotic resistance and use may contribute to those rates through various mechanisms, including lack of clearance of resistant infections following antibiotic treatment, with some of those infections subsequently devolving into sepsis. At the same time, there is limited information on the effect of prescribing of certain antibiotics vs. others on the rates of septicemia and sepsis-related hospitalizations and mortality.

**Methods:**

We used multivariable linear regression to relate state-specific rates of outpatient prescribing overall for oral fluoroquinolones, penicillins, macrolides, and cephalosporins between 2011 and 2012 to state-specific rates of septicemia hospitalization (ICD-9 codes 038.xx present anywhere on a discharge diagnosis) in each of the following age groups of adults: (18-49y, 50-64y, 65-74y, 75-84y, 85 + y) reported to the Healthcare Cost and Utilization Project (HCUP) between 2011 and 2012, adjusting for additional covariates, and random effects associated with the ten US Health and Human Services (HHS) regions.

**Results:**

Increase in the rate of prescribing of oral penicillins by 1 annual dose per 1000 state residents was associated with increases in annual septicemia hospitalization rates of 0.19 (95% CI (0.02,0.37)) per 10,000 persons aged 50-64y, of 0.48(0.12,0.84) per 10,000 persons aged 65-74y, and of 0.81(0.17,1.40) per 10,000 persons aged 74-84y. Increase by 1 in the percent of African Americans among state residents in a given age group was associated with increases in annual septicemia hospitalization rates of 2.3(0.32,4.2) per 10,000 persons aged 75-84y, and of 5.3(1.1,9.5) per 10,000 persons aged over 85y. Average minimal daily temperature was positively associated with septicemia hospitalization rates in persons aged 18-49y, 50-64y, 75-84y and over 85y.

**Conclusions:**

Our results suggest positive associations between the rates of prescribing for penicillins and the rates of hospitalization with septicemia in US adults aged 50-84y. Further studies are needed to better understand the potential effect of antibiotic replacement in the treatment of various syndromes, including the potential impact of the recent US FDA guidelines on restriction of fluoroquinolone use, as well as the potential effect of changes in the practices for prescribing of penicillins on the rates of sepsis-related hospitalization and mortality.

## Background

Rates of hospitalization with septicemia, sepsis, associated mortality and monetary costs have been rising rapidly during the past decades in the US [1–4]. While changes in diagnostic practices contributed to the rise in the rates of hospitalization with septicemia/sepsis in the diagnosis [5, 6], those changes cannot fully explain that rise in hospitalization rates, particularly prior to 2010 [7]. Indeed, trends in the rates of hospitalization with any diagnosis of sepsis between 2003 and 2009 closely resembled the trends in the rates of hospitalizations that involved infection and the use of mechanical ventilation (Figure 1 in [7]). Moreover, rates of hospitalization with severe sepsis in the diagnosis were undergoing robust growth between 2008 and 2012, and the percent of those hospitalizations that involved multiple organ failure was also rising [8], suggesting genuine growth in the volume of hospitalization involving severe sepsis.

Delineation of the factors behind the growth in the volume of hospitalization involving severe bacterial infections [7, 8], as well as the high rates of those hospitalizations [2] should help inform mitigation efforts. Antibiotic resistance and use contribute to septicemia/sepsis hospitalization and mortality rates [9]. The relation between infections with antibiotic-resistant bacteria and survival for sepsis, including the effect of initially appropriate antibiotic therapy (IIAT) is suggested by a number of studies, e.g. [10, 11]. However, less is known about the relation between levels of antibiotic use, as well as prevalence of antibiotic resistance and rates of hospitalization for septicemia/sepsis. Antibiotic resistance can facilitate the progression to severe disease when infections not cleared by antibiotics prescribed during outpatient and/or the inpatient treatment subsequently devolve into a more severe state, including sepsis. For example, antibiotic resistance in Enterobacteriaceae, including fluoroquinolone resistance in *Escherichia coli* was found to be associated with a more severe presentation in urinary tract infections (UTIs) [12, 13], while prevalence of fluoroquinolone resistance in *E. coli* was strongly correlated with rates of septicemia hospitalization and sepsis mortality in adults in different US states [9]. In a study of cases of *E. coli* bacteremia in England, urogenital infection had been treated in 310/891 (34.8%) cases in the 4 weeks preceding bacteremia and this sub-population differed very significantly in its antibiotic resistances [14], suggesting treatment failure due to presence of antibiotic resistance prior to the onset of bacteremia. In particular, prevalence of co-amoxiclav resistance in bacteremia outcomes in England, particularly *E. coli* bacteremia is very high [15] (e.g. more than twice as high as prevalence of co-amoxiclav resistance in *E. coli*-related urinary tract infections [16]), and use of co-amoxiclav [17], both in the hospital and the primary care settings, may contribute to the incidence of co-amoxiclav resistant *E. coli* infections/colonization and the associated bacteremia outcomes. Antibiotic use is one important driver of the prevalence of antibiotic resistance [18–22] and thus may contribute to sepsis incidence. Antibiotic use has been implicated as a cause of resistance not only to the drug class used, but also to other drugs. For example, fluoroquinolone use and resistance was found to be associated with methicillin-resistant *S. aureus* (MRSA) infections [23–25] and extended-spectrum beta-lactamese (ESBL) producing infections [26, 27], with some of those infections leading to sepsis outcomes, while amoxicillin use was found to be associated with trimethoprim resistance in Enterobacteriaceae in England [18], with trimethoprim-resistant urinary tract infections (UTIs) leading to bacteremia outcomes [19]. While causality is not conclusively proven in these studies, it is plausible given the fact that resistance to different drug classes tends to cluster in bacterial populations, leading to the phenomenon of co-selection [28, 29].

Earlier work has shown a relationship, including spatial correlations between the rates of antibiotic consumption and prevalence of antibiotic resistance, e.g. [18–22]. At the same time, there is limited information on the relationship between prescribing for different antibiotic types/classes and rates of septicemia/sepsis hospitalization, including differences in the effect of prescribing of certain antibiotics vs. others on the rates of septicemia/sepsis. Moreover, those relationships/effects may be context-specific and influenced by various factors such as the use of other antimicrobials and prevalence of cross-resistance [27, 30], local patterns of transmission/acquisition of antibiotic-resistant infections, demographic and climatic differences, etc. This study examines (quantifies) the contribution of prescribing for different antibiotic classes to the rates of hospitalization with septicemia using population-level data in the US. We use a regression framework to relate the state-specific rates of outpatient prescribing of oral fluoroquinolones, penicillins, macrolides, and cephalosporins in the US CDC Antibiotic Patient Safety Atlas data [31] to state-specific rates of hospitalization with septicemia (ICD-9 codes 038.xx present on the discharge diagnosis) in different age groups of adults recorded in the Healthcare Cost and Utilization Project (HCUP) data [32], adjusting for additional factors that may affect septicemia hospitalization rates. The framework for the analyses in this paper is similar to the framework for the analyses in our parallel study on the relation between antibiotic prescribing and sepsis mortality [33]. The goal of such analyses is to provide further evidence about the relation between prescribing of certain antibiotics and rates of sepsis-related outcomes, which should help inform antibiotic prescribing policies with the aim of reducing the burden of septicemia and sepsis.

## Methods

### Data

We used data between 2011 and 2012 on counts of hospitalization with a septicemia diagnosis (both primary and contributing, [*ICD-9*] codes 038.xx on the discharge diagnosis) from the State Inpatient Databases of the Healthcare Cost and Utilization Project (HCUP), maintained by the Agency for Healthcare Research and Quality (AHRQ) through an active collaboration [32]. We will henceforth call these hospitalizations septicemia hospitalizations, even though some of them may involve low levels of bloodstream bacterial infection. The database [32] contains hospital discharges from community hospitals in participating states. Forty-two states reported septicemia hospitalization data between 2011 and 2012 for each of the five adult age groups included in our analyses: 18-49y, 50-64y, 65-74y, 75-84y, 85 + y. Those states are Alaska, Arkansas, Arizona, California, Colorado, Connecticut, Florida, Georgia, Hawaii, Iowa, Illinois, Indiana, Kansas, Kentucky, Louisiana, Massachusetts, Maryland, Minnesota, Missouri, Montana, North Carolina, North Dakota, Nebraska, New Jersey, New Mexico, Nevada, New York, Ohio, Oklahoma, Oregon, Rhode Island, South Carolina, South Dakota, Tennessee, Texas, Utah, Virginia, Vermont, Washington, Wisconsin, West Virginia, and Wyoming. Aggregate state-level hospitalization data were used in the analyses, with no informed consent from the participants sought.

We extracted data on the annual state-specific per capita rates of outpatient oral prescribing for four classes of antibiotics: fluoroquinolones, penicillins, macrolides, and cephalosporins in 2011 and 2012 from the US CDC Antibiotic Patient Safety Atlas [31]. Average annual state-specific outpatient prescribing rates, namely the annual number of oral antibiotic prescriptions dispensed to outpatients in community pharmacies per 1000 state residents [31] for each class of antibiotics between 2011 and 2012 were then estimated as the average for the corresponding rates in 2011 and 2012.

Annual state-specific population estimates in each age group of adults were obtained as the yearly July 1 population estimates in [34]. Data on median household income for US states between 2011 and 2012 were extracted from [35]. Data on average minimal daily temperature for US states were obtained from [36]. Data on the percent of state residents in different age groups who lacked health insurance were extracted form the US Census Bureau database [37].

### Regression model

For each age group of adults (18-49y, 50-64y, 65-74y, 75-84y, 85 + y), we applied a mixed-effect multivariable regression model to adjust for various factors that affect the relation between prescribing of different antibiotics and rates of septicemia hospitalization. The dependent variable for each regression model (one for each age group) was the state-specific rate of septicemia hospitalization in the given age group between 2011 and 2012 [32]. The covariates were the state-specific outpatient prescribing rates (per 1000 state residents) for oral fluoroquinolones, penicillins, macrolides, and cephalosporins between 2011 and 2012 [31], the state-specific median household income, the state-specific average minimal daily temperature, and percentages of state residents in the given age group who were African American, as well as those who lacked health insurance (for age groups under 65 years). We note that temperature may influence bacterial growth rates and/or transmission mediated effects [38, 39], which in turn may affect both the prevalence of antibiotic resistance and the acquisition/severity of bacterial infections. We also note that African Americans have elevated rates of sepsis compared to other races [40]. To adjust for additional factors not accounted for by the covariates used in the model, we include random effects for the ten Health and Human Services (HHS) regions in the US. Specifically, for each state *s* that reported the corresponding septicemia hospitalization data between 2011 and 2012 in [32], let *HR*(*s*) be the state-specific rate of hospitalizations with septicemia in the given age group per 10,000 residents, *A*_*i*_(*s*) (*i* = 1, .., 4) be the state-specific average annual outpatient prescribing rates for oral antibiotics between 2011 and 2012, per 1000 state residents (of all ages), for the four studied classes of antibiotics (thus *A*_1_(*s*) denotes the rate of prescribing of oral fluoroquinolones, etc.); *I*(*s*) be the median state-specific household income (in $1000 s) between 2011 and 2012; *MT*(*s*) be the average *minimal* daily temperature (°*F*) between 2005 and 2011 for the state; *AA*(*s*) be the percent of African Americans among the state residents in a given age group between 2011 and 2012; *LHI*(*s*) be the average annual age-specific percent of state residents who lacked health insurance between 2011 and 2012 (for non-elderly age groups); *α*(*s*) be the random effect for the corresponding HHS region, and *ε* be the residual. Then:

1 $$ HR(s)={\beta}_0+\sum \limits_{i=1}^4{\beta}_i\bullet {A}_i(s)+{\beta}_5\bullet I(s)+{\beta}_6\bullet MT(s)+{\beta}_7\bullet AA(s)+..+{\beta}_8\bullet LHI(s)+\alpha (s)+\varepsilon $$

This regression was performed five separate times, once for the outcome in each age group, with the health insurance and percent African-American covariates being age-group-specific, and the others being the same between regressions. The random effect was estimated independently in each regression.

## Results

Table 1 shows, for the different age groups of adults, the mean (standard error) for the annual rates of hospitalization with septicemia per 10,000 individuals in a given age group between 2011 and 2012 for the 42 states reporting the corresponding data (Methods). Those rates increase rapidly with age, from a mean of 16.3 (standard error 4.3) for individuals aged 18-49y to a mean of 339.7 (96.4) for individuals aged over 85y. Table 1 also shows, for the different age groups, the correlations between the state-specific rates of septicemia hospitalization in a given age group between 2011 and 12 and the state-specific rates of outpatient prescribing of all oral antibiotics between 2011 and 2012. Those correlations are fairly high, peaking at 0.51(95% CI (0.24,0.70)) for persons aged 50-64y. We note that correlations between the state-specific rates of outpatient prescribing of all oral antibiotics and rates of sepsis mortality in different age groups are even higher (compare with Table 1 in [33]).

**Table 1:** Mean (standard error) for the annual rates of hospitalization with septicemia per 10,000 individuals in different age groups between 2011 and 2012 for the 42 states reporting the corresponding data (Methods), and the correlations (with 95% CIs; boldface indicates statistical significance) between the state-specific septicemia hospitalization rates in different age groups between 2011 and 12 and the state-specific rates of prescribing of all oral antibiotics between 2011 and 2012

Age	Average annual rates of septicemia hospitalization per 10,000 (standard error)	Correlation between septicemia hospitalization rates and rates of overall outpatient oral antibiotic prescribing (95% CI)
18-49y	16.3 (4.3)	**0.44 (0.15,0.65)**
50-64y	54.5 (12)	**0.51 (0.24,0.70)**
65-74y	118.0 (24)	**0.49 (0.21,0.69)**
75-84y	214.4 (46.9)	**0.41 (0.12,0.63)**
85 + y	339.7 (96.4)	0.30 (−0.01,0.55)

Partitioning the relationship between the rates of antibiotic prescribing and rates of septicemia hospitalization by antibiotic class is challenging, in part because of the high positive correlations between the rates of outpatient prescribing for different antimicrobial classes at the state level. Table 2 presents the correlations between the state-specific rates of outpatient prescribing for the different classes of oral antibiotics (fluoroquinolones, penicillins, macrolides, and cephalosporins) in [31]. All the correlations in Table 2 are high, with point estimates above 0.76 for each pair of classes of antibiotics.

**Table 2:** Correlations (with 95% CIs; boldface indicates statistical significance) between the state-specific rates of outpatient prescribing of fluoroquinolones, penicillins, macrolides, and cephalosporins in [31] between 2011 and 2012

	Fluoroquinolones	Penicillins	Macrolides	Cephalosporins
Fluoroquinolones	1			
Penicillins	**0.8 (0.66,0.89)**	1		
Macrolides	**0.88 (0.79,0.94)**	**0.83 (0.7,0.91)**	1	
Cephalosporins	**0.76 (0.59,0.86)**	**0.82 (0.69,0.9)**	**0.82 (0.68,0.9)**	1

Table 3 presents the estimates (regression coefficients) for the multivariable regression model (eq. 1) that relates the state-specific rates of outpatient prescribing for the four studied classes of oral antibiotics, the median household income, average minimal daily temperature, and percentages of state residents in a given age groups who were African American, as well as those who lacked health insurance (for non-elderly age groups) to the state-specific septicemia hospitalization rates in different age groups of adults between 2011 and 2012 [32]. Table 3 suggests that an increase in the rate of prescribing of oral penicillins by 1 annual dose per 1000 state residents was associated with an increase of 0.19(95% CI (0.02,0.37)) in the annual septicemia hospitalization rate per 10,000 persons aged 50-64y, an increase of 0.48(0.12,0.84) in the annual septicemia hospitalization rate per 10,000 persons aged 65-74y, and an increase of 0.81(0.17,1.40) in the annual septicemia hospitalization rate per 10,000 persons aged 74-84y. Increase by 1 in the percent of state residents in a given age group who are African American was associated with increases in annual septicemia hospitalization rates of 2.3(0.32,4.2) per 10,000 persons aged 75-84y, and of 5.3(1.1,9.5) per 10,000 persons aged over 85y. Additionally, average minimal daily temperature was positively associated with septicemia hospitalization rates in persons aged 18-49y, 50-64y, 75-84y and over 85y.

**Table 3:** Regression coefficients (with 95% CIs; boldface indicates statistical significance) in eq. 1 for the different covariates in the model given by eq. 1 for different age groups. The coefficients for the different antibiotic classes estimate the change in the annual septicemia hospitalization rates (per 10,000 individuals in a given age group) when the annual rate of outpatient prescribing of the corresponding oral antibiotic class (per 1000 residents) increases by 1. ND = not done because persons > 64 years old are eligible for Medicare

	Aged 18-49y	Aged 50-64y	Aged65-74y	Aged 75-84y	Aged 85 + y
Fluoroquinolones (prescription per 1000 residents/y)	0.08 (−0.07,0.22)	0.18 (− 0.15,0.51)	0.36 (− 0.3,1)	0.7 (− 0.45,1.9)	1.3 (−1.1,3.7)
Penicillins (prescription per 1000 residents/y)	0.04 (− 0.03,0.12)	**0.19 (0.02,0.37)**	**0.48 (0.12,0.84)**	**0.81 (0.17,1.4)**	1.2 (−0.14,2.5)
Cephalosporins (prescription per 1000 residents/y)	−0.02 (− 0.09,0.05)	−0.03 (− 0.19,0.13)	−0.08 (− 0.42,0.25)	−0.28 (− 0.88,0.31)	−0.69 (− 1.9,0.51)
Macrolides (prescription per 1000 residents/y)	− 0.03 (− 0.11,0.04)	−0.12 (− 0.29,0.04)	−0.26 (− 0.6,0.08)	−0.39 (− 0.99,0.22)	−0.55 (− 1.8,0.7)
Median household income ($1000)	− 0.09 (− 0.29,0.1)	−0.17 (− 0.58,0.24)	0.31 (− 0.5,1.1)	1.2 (− 0.24,2.6)	**3.3 (0.42,6.2)**
Average minimal daily temperature (°*F*)	**0.2 (0,0.39)**	**0.57 (0.12,1)**	0.78 (−0.14,1.7)	**1.6 (0.03,3.2)**	**3.9 (0.59,7.3)**
Percent African Americans	−0.05 (− 0.22,0.12)	0.15 (− 0.28,0.59)	0.79 (− 0.23,1.8)	**2.3 (0.32,4.2)**	**5.3 (1.1,9.5)**
Percent lacking health insurance	0 (−0.25,0.24)	− 0.16 (− 0.91,0.59)	ND	ND	ND

## Discussion

While antimicrobial use can contribute to the rates of hospitalization with septicemia and sepsis (see the Introduction), the strength of the relation between antibiotic prescribing rates and rates of septicemia/sepsis hospitalization may vary with antibiotic types/classes and depend on a number of factors including patterns of resistance to different antibiotics. In this study, we use a mixed-effect multivariable model to examine the relation between the rates of prescribing of different classes of oral antibiotics and the rates of septicemia hospitalization in different age subgroups of adults in the US context. Our analyses suggest the association between the state-level rates of outpatient prescribing of oral penicillins and rates of septicemia hospitalization in persons aged 50–64 years, 65–74 years, and 75–84 years. We note that our companion paper [33] has shown an association between the state-level rates of outpatient prescribing of penicillins and rates of septicemia mortality in older adults. We also note that earlier work has shown a relationship, including spatial correlations between the rates of antibiotic prescribing and antibiotic resistance, e.g. [18–22]. The findings in this paper and in our parallel study [33], as well as the high prevalence of resistance to penicillins in both the Gram-negative and Gram-positive bacteria (e.g. [30, 41, 42]) support the notion that the combination of the use of penicillins and resistance to penicillins is associated with the rates of sepsis hospitalization and mortality in older US adults. Further studies of various related phenomena, including patterns of resistance to different antibiotics for different bacterial infections, and individual-level analyses of the effect of prescribing of different antibiotics in the treatment of various syndromes on subsequent sepsis-related outcomes are needed to better understand the potential effect of antibiotic, particularly penicillin replacement in the treatment of various syndromes on the rates of sepsis hospitalization and mortality. Additionally, we have found that prevalence of African Americans among older adults is associated with rates of septicemia hospitalization, which agrees with earlier findings that rates of sepsis hospitalization among African Americans are elevated [40]. Our work also suggests that average minimal daily temperature is positively associated with the rates of septicemia hospitalization in four out of five age groups of adults, which resonates with earlier findings about the association between temperature and antibiotic resistance [38, 39].

In addition to the associations between the rates of prescribing of penicillins and rates of septicemia hospitalization in persons aged 50-84y, our analyses have found suggestive evidence of an association between the rates of fluoroquinolone prescribing and rates of sepsis hospitalization, but in no age group did the confidence bounds for this association exclude zero. Additionally, our earlier work [9] has shown associations between prevalence of resistance to fluoroquinolones in *E. coli* and the rates of both septicemia hospitalization and sepsis mortality in different age groups of US adults, with prevalence of fluoroquinolone resistance in *E. coli*-related UTIs in the US being high [27, 30]. We note the high frequency of fluoroquinolone use in the treatment of UTIs in the US, both in women and men [43, 44] despite the high prevalence of fluoroquinolone resistance in UTIs – for example, compare the Veterans Affairs data on antibiotic use [43] vs. antibiotic resistance [30] for both fluoroquinolones and trimethoprim. Recently, US FDA has recommended the restriction of fluoroquinolone use for certain conditions (such as uncomplicated UTIs) due to potential adverse effects [45]. However, adherence to earlier recommendations [46] for avoidance of fluoroquinolones in the treatment of uncomplicated UTIs was limited [44]. We note that the UK has updated its antibiotic prescribing guidelines for UTIs, with nitrofurantoin generally recommended as the first-line option ([15], p. 36), and that a good amount of replacement of trimethoprim by nitrofurantoin in the treatment of UTIs in the UK took place during the recent years [15].

Our results about the relation between the use of penicillins and rates of sepsis-related outcomes are similar to some of the findings from the UK. Our parallel study suggests associations between the rates of prescribing of penicillins and rates of *E. coli* and MSSA bacteremia in England [33]. In the UK, outpatient amoxicillin prescribing rates are high (Figure 3.4 in [15]), and amoxicillin use is associated with the prevalence of trimethoprim resistance [18], while trimethoprim use in turn is associated with the rates of UTI-related *E. coli* bacteremia [19]. The latter association is presumably mediated by the treatment of trimethoprim-resistant UTIs with trimethoprim. Another antibiotic relevant to the growth in the rates of *E. coli*-related bacteremia in England [16, 22] is amoxicillin-clavulanate (co-amoxiclav). Levels of *E. coli* and *Klebsiella*-related bacteremia were continuing to rise in England after 2006 while reduction in fluoroquinolone and cephalosporin use was taking place [16, 22]. Amoxicillin-clavulanate prescribing in England increased significantly between 2006 and 2011 [47], and incidence of bacteremia with *E. coli* strains resistant to co-amoxiclav began to increase rapidly after 2006 (Figure 4 in [17]), with co-amoxiclav resistance in *E. coli* bacteremia exceeding 40% in 2014 [15, 16]. Additionally, prevalence of co-amoxiclav resistance in bloodstream *E. coli*-associated infections in England is notably higher than the prevalence of co-amoxiclav resistance in *E. coli*-associated UTIs (compare Figure 2.1 in [16] vs. Figure 2.7 in [16]), which is suggestive of the role of the use of co-amoxiclav and other penicillins in secondary care (whose volume is very high, Table 3.4 in [15]) in the burden of *E. coli*-related bacteremia. Those findings from the UK support both the role of penicillin use in the burden of sepsis-related outcomes and the fact that data on resistance patterns for different antibiotics in different bacteria contributing to various syndromes should be considered when new antibiotic prescribing practices are being phased in. There is evidence that reduction in fluoroquinolone and cephalosporin prescribing in England was associated with decreases in the rates of *C. difficile* and MRSA infections [25, 48, 49]. At the same time, the parallel increase in penicillin prescribing appears to have helped sustain the growth in the rates of other outcomes associated with bacterial infections, particularly *E. coli* bacteremia.

Cephalosporin and macrolide prescribing had suggestive negative associations with the rates of sepsis hospitalization in our analysis, but again all confidence intervals included zero. If (like all models) this model is somewhat mis-specified, competition with antibiotics that are more likely to promote sepsis outcomes could bias downward the strength of the association between the use of certain antibiotics and the rates of sepsis hospitalization. The negative point estimates for the contribution of cephalosporins and macrolides could have been affected by this phenomenon, particularly competition with penicillins and possibly fluoroquinolones for which there is high prevalence of resistance in different types of infection, e.g. [27, 30, 41, 42]. Additionally, various sources of noise, such as the fact that we related antibiotic prescribing rates in the population overall to rates of septicemia hospitalization in different age groups, might have reduced the precision of our estimates. We note that prevalence of cephalosporin resistance and the frequency of extended-spectrum beta-lactamase (ESBL)-producing bacterial infections, particularly for Gram-negative bacteria is growing [50]. Macrolide use could potentially contribute to the rates of sepsis as macrolides are commonly prescribed for the treatment of certain syndromes that are major causes of sepsis, notably respiratory diseases, both chronic [51] and acute, including pneumonia [52]. On the other hand, macrolides are used relatively infrequently in the treatment of urinary tract and gastrointestinal infections. A UK study found that for a high proportion of Gram-negative bacteremia, the main foci of infection were either urinary tract or abdomen/biliary tract [53]. Overall, the potential benefits of antibiotic stewardship for penicillins may be the most important findings of this paper, while options for antibiotic replacement require further investigation.

Our study has some additional limitations. The study is ecological, but for exposures such as antibiotic use, this may be the best design because effects of antibiotic use on resistance go beyond the treated individual to those with whom transmission (directly or indirectly is possible). The antibiotic-sepsis incidence associations we find estimate causal effects only if the model is well-specified and all confounders are accounted for in the analysis. We have included several relevant demographic and geographic variables – income and average minimal daily temperature, percentages of residents who are African American, and those who lack health insurance – with known or hypothesized associations with both antibiotic use and sepsis. We have used age stratification in the outcome to deal with confounding by age composition. Nevertheless, there may be misspecification and residual confounding (such as geographic differences in coding practices for septicemia). To adjust for potential effects of confounding/model misspecification, we have included random effects for the ten US Health and Human Services regions, which led to improvements in model fits. We also note that reverse causality associated with antibiotic treatment for sepsis is unlikely to have a significant contribution to our results because we are considering the relation between *outpatient* prescribing rates and septicemia hospitalizations. Nonetheless, further studies using other methods and in other populations (including individual-level analyses) are needed to ascertain the consistency of our findings. While the HCUP data utilized in our study generally cover about 97% of all community hospitalizations in the US [32], state-specific variability in the proportion of septicemia hospitalizations that are covered by the HCUP data is possible. Diagnostic practices for septicemia vary by state. It is uncertain whether states with higher antibiotic prescribing rates have more inclusive criteria for a septicemia diagnosis (boosting the association between antibiotic prescribing rates and septicemia hospitalization rates), or vice versa. For example, California has relatively low antibiotic prescribing rates [54] and apparently more inclusive criteria for a septicemia/sepsis diagnosis that translate into lower case fatality rates for such hospitalizations compared to the national average, e.g. [55]. On the other hand, some of the states with low antibiotic prescribing rates, possibly outside the Northeastern US, may have more strict criteria for a septicemia/sepsis diagnosis compared to the national average. Finally, data on outpatient antibiotic prescribing in the whole population [31] were used in the regression model for which the outcomes were age-specific rates of septicemia hospitalization [32]. We expect that those sources of noise/incompatibility should generally reduce precision and bias toward null results rather than create spurious associations.

## Conclusions

We believe that despite some limitations, our findings indicate a possible causal association between the use of penicillins, and the rates of sepsis hospitalization in US adults aged between 50-84y, suggesting the potential benefits of antibiotic stewardship. We hope that this ecological study would lead to further investigations of the relation between antibiotic prescribing practices and sepsis in different contexts, including individual-level analyses of the relation between prescribing of different antibiotics for the treatment of various syndromes and subsequent septicemia/sepsis outcomes. Such analyses are needed to inform antibiotic prescribing guidelines, including the potential replacement of some antibiotics, particularly penicillins in older adults by other antibiotics in the treatment of certain syndromes with the aim of mitigating the rates of septicemia, sepsis, and the associated mortality. Finally, we believe that a comprehensive, long-term approach for controlling the rates of severe outcomes associated with bacterial infections, including sepsis should include not only the adoption of appropriate antibiotic prescribing practices but also the introduction of new antibiotics [56].

## Data Availability

The datasets generated and analyzed during the current study are available in the data repositories described in refs. [31, 34–37], as explained in the Methods section; those datasets can be downloaded through the corresponding URLs provided within those references. The only exception is the septicemia hospitalization data that were provided by the State Inpatient Databases of the Healthcare Cost and Utilization Project (HCUP) (ref. [32]) for the purposes of this study. Those data are available from the authors upon reasonable request and with permission of the Healthcare Cost and Utilization Project.

## Abbreviations

*E. coli*: *Escherichia coli*
HCUP: Healthcare Cost and Utilization Project
US FDA: United States Food and Drug Administration
US HHS: United States Health and Human Services
UTI: urinary tract infection
